# A Machine for Inducing Artificial Respiration

**Published:** 1908-04-11

**Authors:** 


### NEW APPLIANCES AND THINGS MEDICAL.

[We shall be glad to receive at our Office, 23 & 29 Southampton Street, Strand, London, W.C., from the manufacturers, specimens of all new preparations and appliances which may be brought out from time to time.]

### A MACHINE FOR INDUCING ARTIFICIAL RESPIRATION.

Professor George Poe, an American scientist, has invented and patented a machine for inducing artificial respiration, which, if it fulfils the claims of its designer, will be an invaluable apparatus in the hands of the physician.

The machine has recently been submitted to several tests, and if the reports of these tests may be accepted as reliable the inventor has certainly made an interesting discovery. It was really an accident that led Professor Poe to devise his machine. In the year 1876 he succeeded in resuscitating a rat, which was supposed to be dead, by the simple process of pumping oxygen into its lungs. With this success as a basis to work upon, he was encouraged to continue investigations and experiments along the same line, and these have resulted in the artificial respirator.

The apparatus is modelled as nearly as possible after nature. Experience had proved to the inventor that in order to revive persons drowned, suffocated, or whose "death" had been caused by anæsthetics, it was necessary to remove the poisonous gases in the lungs, replacing them by oxygen, and it was to accomplish this double purpose that his present machine was constructed. The instrument consists of two small cylinders, each having an inlet and an outlet, plungers within these cylinders working simultaneously. Tubes lead from each of the cylinders, to be connected to the nostrils or mouth of the patient. The inlet of one cylinder is connected with a suitable supply of oxygen, and the outlet of the other cylinder discharges directly into the atmosphere. The plungers are driven by hand, and timed to correspond to normal respiratory movements, and this action of the plungers in one movement draws the gases from the lungs into one cylinder, while the next movement forces oxygen from the second cylinder into the lungs. It is at once a simple yet ingenious device which anyone can quickly learn to manipulate.

One of the most striking resuscitating tests to which the apparatus was applied was that of a rabbit. The animal was given, by a physician appointed by a committee of experts, two grains of morphine and four ounces of ether. Every test known to science was then made, and the rabbit was declared "dead" by all present so far as medical science could tell. The tubes were then applied to its nostrils, and the plungers operated. Within three minutes the rabbit was breathing in a natural manner, and in six minutes was running about the room. Another subject tested was a dog which for forty minutes had been smothered in acetylene, one of the most deadly of poisonous gases. This dog was revived within a very short period, and showed absolutely no effects from either the smothering or the resuscitation.

Professor Poe claims that the following is entirely new in his invention, "a charging cylinder for delivering oxygen, a discharging cylinder for withdrawing carbonic acid gas, an inlet and an outlet for each cylinder, and a hand piece in communication with the inlet of one cylinder and the outlet of the other, said hand piece including independent chambers having valved communication, and service pipes leading from the respective chambers."

In the case of prevention of death at birth through infantile asphyxia, the machine should certainly prove valuable. Much of the danger of death from the administration of too large anæsthetic doses would be eliminated, as the machine will sustain artificial respiration as long as may be necessary. A man in a drunken stupor could be quickly sobered by using the machine to quicken his respiration, and, as death by freezing is simply a form of asphyxiation, it could be avoided by the use of the apparatus. Physicians are often called upon to assure a family that a patient is absolutely dead and not in a state of suspended animation. The application of the instrument to the patient would positively testify whether life was extinct or not. Then the machine would be valuable to all life-saving stations, and, indeed, to everybody and to every institution whose duties are such that they are called upon to restore animation to partially suffocated persons.

